# An evidence-based recommendation on bed head elevation for mechanically ventilated patients

**DOI:** 10.1186/cc10135

**Published:** 2011-04-11

**Authors:** Barbara S Niël-Weise, Petra Gastmeier, Axel Kola, Ralf P Vonberg, Jan C Wille, Peterhans J van den Broek

**Affiliations:** 1 Leiden University Medical Center, Dutch Working Party Infection Prevention, C7-130, Postbus 9600, 2300 RC Leiden, Germany; 2 Institute of Hygiene and Environmental Medicine, Charité-University Medicine Berlin, Hindenburgdamm 27, 12203 Berlin, Germany; 3 Institute for Medical Microbiology and Hospital Epidemiology, Hannover Medical School, Carl-Neuberg-Straße 1, 30625 Hannover, Germany; 4 CBO, Churchilllaan 11, 3527 GV Utrecht, Germany; 5 Leiden University Medical Center, Department of Infectious Diseases, C5-P, Box 9600, 2300 RC Leiden, Germany

## Abstract

**Introduction:**

A semi-upright position in ventilated patients is recommended to prevent ventilator-associated pneumonia (VAP) and is one of the components in the Ventilator Bundle of the Institute for Health Care Improvement. This recommendation, however, is not an evidence-based one.

**Methods:**

A systematic review on the benefits and disadvantages of semi-upright position in ventilated patients was done according to PRISMA guidelines. Then a European expert panel developed a recommendation based on the results of the systematic review and considerations beyond the scientific evidence in a three-round electronic Delphi procedure.

**Results:**

Three trials (337 patients) were included in the review. The results showed that it was uncertain whether a 45° bed head elevation was effective or harmful with regard to the occurrence of clinically suspected VAP, microbiologically confirmed VAP, decubitus and mortality, and that it was unknown whether 45° elevation for 24 hours a day increased the risk for thromboembolism or hemodynamic instability. A group of 22 experts recommended elevating the head of the bed of mechanically ventilated patients to a 20 to 45° position and preferably to a ≥30° position as long as it does not pose risks or conflicts with other nursing tasks, medical interventions or patients' wishes.

**Conclusions:**

Although the review failed to prove clinical benefits of bed head elevation, experts prefer this position in ventilated patients. They made clear that the position of a ventilated patient in bed depended on many determinants. Therefore, given the scientific uncertainty about the benefits and harms of a semi-upright position, this position could only be recommended as the preferred position with the necessary restrictions.

## Introduction

Ventilator-associated pneumonia (VAP) is defined as pneumonia in a patient on mechanical ventilatory support (by endotracheal tube or tracheostomy) for more than 48 hours. VAP is associated with increased mortality and morbidity in critically ill patients [1].

Aspiration of oropharyngeal secretions and gastric contents containing bacteria is considered an essential step in the pathogenesis of VAP. About 20 years ago, studies using radiolabeled enteral feeding have shown more aspiration of gastric contents in supine patients than in patients in a 45° position [2-4]. None of these studies assessed the effect of a semi-upright position on VAP. An observational study demonstrated that a supine position during the first 24 hours of mechanical ventilation is an independent risk factor for VAP [5]. In contrast to these older studies, a nonexperimental study in 2005 found that only the combination of early, low backrest elevation <30° and severity of illness affected the incidence of VAP [6]. A prospective randomized animal study showed that a semi-upright position reversed the mucus flow in intubated sheep [7].

As a result of the older studies and the paper published by Drakulovic and colleagues in 1999 [8], a semi-upright position for ventilated patients has been promoted to prevent VAP. Besides its benefits, however, a semi-upright position may have disadvantages such as venous stasis in the lower extremities with the risk of venous thromboembolism [9], caudal shift of blood with the risk of hemodynamic instability [10], and the risk of bed sores [6]. In addition, the feasibility of a 45° backrest position in the 'real life' of today's ICUs is debated [11].

The Canadian Critical Care Trials Group, the American Thoracic Society and Infectious Diseases Society of America, and the Centers for Disease Control and Prevention recommend the use of a semi-upright position (30 to 45°), especially in patients receiving enteral feeding [12-14]. Because these recommendations are not based on the findings of a systematic review, a Dutch-German review group decided to summarize the evidence of the benefits and disadvantages of semi-upright positioning in ventilated patients by a systematic review of the literature.

Systematic reviews are the basis of evidence-based recommendations. Next to the scientific evidence, other considerations play a role in formulating recommendations for clinical practice. In the second part of this study, a European expert panel of intensive care specialists developed a recommendation on bed head elevation based on the results of the systematic review and considerations beyond the scientific evidence in a three-round electronic Delphi procedure.

## Materials and methods

### Systematic review

#### Question

Two issues were addressed: should bed head elevation be higher than standard practice, and which degree of bed head elevation does more good than harm? Bed head elevation was defined as the angle of the head of the bed and was expressed in degrees of elevation above horizontal.

#### Outcomes

Primary outcomes were clinically suspected VAP and microbiologically confirmed VAP. Clinically suspected VAP was defined as new or persistent or progressive radiographic infiltrate with at least two of the following criteria: fever; leucopenia or leucocytosis; and purulent tracheal secretions. VAP was microbiologically confirmed when cultures of airway secretions were positive.

Secondary outcomes included mortality, venous thromboembolism, hemodynamic instability, duration of mechanical ventilation, length of ICU stay, decubitus ulcers, patient comfort and patient safety.

#### Searching

Publications were retrieved by searching Medline and the Cochrane Library up to 31 January 2010. Search terms included randomized controlled trial (RCT), backrest elevation and semirecumbent. The complete search strategy can be found in the electronic supplementary material (Additional file 1). In addition, the lists of references for all identified trials were checked for more trials.

#### Selection

We included studies that were planned as a randomized trial or a quasi-randomized trial and were also published as a full paper. The studies had to state the outcome definitions used and had to present sufficient data to be able to calculate the risks in both the treatment group and the control group. No language restrictions were applied. Two reviewers (BSN-W and AK) assessed all titles and abstracts independently to confirm the eligibility of the selected trials. Disagreements were resolved by consensus.

#### Assessment of trial quality

Six reviewers assessed trial quality independently by evaluating each study to determine concealment of treatment allocation, double-blinding, completeness of follow-up, use of intention-to-treat analysis, selective reporting of events and premature discontinuation of the trial due to benefit. Central randomization, sealed envelopes or a similar method was assumed to yield adequate randomization. The description of dropouts was considered adequate if the number of patients lost and the reasons why patients were lost to follow-up were reported according to treatment allocation. Disagreements were resolved by consensus.

#### Data extraction, analysis and quality of evidence

The systematic review was performed according to PRISMA guidelines [15]. Data on the study population, interventions and outcomes were independently extracted and cross-checked by six reviewers. Only trial data related to the question posed in the review were considered.

For the dichotomous outcomes we calculated the overall relative risk (RR) with a 95% confidence interval (CI) by means of Review Manager (Version 5; Cochrane Collaboration, Oxford, UK), using the standard random-effects method of DerSimonian and Laird [16]. When appropriate, meta-analyses were undertaken using a random-effects model to calculate pooled estimates and their 95% CIs. Subgroup meta-analyses were planned according to feeding via gastric tubes or not; continuous subglottic suctioning of oropharyngeal secretions via modified tubes or not; use of selective digestive tract decontamination or not; use of antacids or not; and presence of hiatal hernia or not. Baseline risks were defined as the risk for a certain outcome in the control arm of the individual trials - that is, the risk for a certain outcome without treatment (total number of patients with a certain outcome in the control group/total number of patients in the control group). Publication bias was examined by visual inspection of a funnel plot. The quality of the available evidence for each outcome was assessed by the GRADE method [17].

### Development of a recommendation by an electronic Delphi consensus process

#### Participants

European experts in intensive care medicine who published an article in the field of the prevention of VAP, who were a member of a national or international platform/research group on VAP prevention, or who were specialized in VAP prevention were personally invited for study participation. Furthermore, the study was announced on the website of the European Society of Intensive Care Medicine [18] and in the society's Newsletter, offering every member of the society the opportunity to participate.

#### Procedure

Three Delphi rounds were performed by a web-based electronic form in an anonymous fashion. Each round lasted about 3 weeks. After each round the participants received a feedback report from the previous round. Nonresponders were excluded from the next Delphi round.

The first expert consultation was designed to give them the opportunity to comment on the results of the systematic review, to identify considerations beyond the scientific evidence of importance for the formulation of the recommendation, and to check first opinions about the phrasing of a recommendation. A number of possible considerations were proposed by the authors - that is, feasibility of a semi-upright position, external validity of the scientific evidence, difficulty in measuring adherence to a semi-upright position, costs, and the use of a semi-upright position in bundles for the prevention of VAP. These considerations were presented to the participants, who were asked to indicate whether they agreed or disagreed by marking one of the listed answers; to comment their answers in comment boxes; and to add more considerations. Differently phrased recommendations were presented to indicate agreement or disagreement as described above. The experts could formulate their own recommendation if their preferred recommendation was not phrased.

The second expert consultation depended completely on the results of the first Internet consultation.

Based on the results of the systematic review and the results of the first and second rounds, the investigators proposed a recommendation and motivation for this recommendation (rationale). In the third Internet round, the participants were asked to state whether they agreed or disagreed with the proposed recommendation and its rationale.

## Results

### Systematic review

#### Selection

Two hundred and eight potentially relevant studies were initially identified by our search. Three studies fulfilled the selection criteria and were included in the review [8,19,20]. The flow diagram showing the steps we followed to identify the RCTs fulfilling the inclusion criteria of our systematic review can be found in Additional file 1.

#### Quality assessment of trials

All three trials clearly concealed patient allocation, adequately described dropouts and did not report events in a selective way. A single trial blinded the investigators responsible for VAP diagnosis [20] and used intention-to-treat analysis [20]. In one trial, 46% of the randomized patients did not complete the trial [19]. One trial stopped before the intended sample size was reached because of benefit [8].

#### Data extraction, analysis and body of evidence

Data on study populations, interventions and outcome definitions are shown in Table 1. Three RCTs compared 45° bed head elevation (treatment group) with respectively 25° [19], 10° [20], or 0° [8] elevations (control groups) in adult ventilated ICU patients with a mean ventilation time varying from 4 days [19] to 7 days [8]. Patients with hemodynamic instability, pelvis trauma, (recent) abdominal surgery or neurosurgery, and severe obesity were excluded.

**Table 1:** Study populations, interventions and definition of VAP

Study	Study participants (number of patients randomized)	Treatment (T) and Control (C) groups (number of patients analyzed)	Duration of ICU ventilation	Outcomes	Baseline risks, %	End of study protocol
Drakulovic and colleagues [8]	Respiratory and medical ICU patients (90). Excluded: previous endotracheal intubation (<30 days); recent abdominal surgery; recent neurosurgical intervention; hemodynamic instability	T (39), 45°; C (47), 0°. Correctness of the position was checked once a day	Mean: T, 145 hours (SD 149); C, 171 hours (SD 167)	Clinically suspected VAP, defined as new and persistent infiltrate on chest radiography and at least two of the following three criteria: fever; leucopenia or leucocytosis; purulent tracheal secretions: T, 3/39; C, 16/47	Clinically suspected VAP: 34%	End of study protocol: (1) first weaning trial, (2) extubation, (3) death, (4) permanent change in body position for more than 45 minutes
				Microbiologically confirmed VAP, defined as clinical suspicion and positive ETS, BAL or PSB: T, 2/39; C, 11/47	Microbiologically confirmed VAP: 23%	Follow-up for an additional 72 hours after the study end point has been reached
				ICU mortality: T, 7/39; C, 13/47	For ICU mortality: 28%	
Keeley [19]	Adult ventilated patients with no contraindications for raised head of bed (56). Excluded: previous endotracheal intubation (<30 days); recent abdominal surgery with vacuum dressing; severe obesitas; hemodynamic instability; renal replacement therapy; pregnancy; spinal surgery or trauma	T (17), 45°; C (13), 25°. Authors did not report whether correctness of the position was checked during the study	Mean^a^: T, 3.8 days; C, 5.1 days	Clinically suspected VAP, defined as new and persistent infiltrate on chest radiography and at least two of the following three criteria: fever; leucopenia or leucocytosis; purulent tracheal secretions: T, 1/17; C, 2/13	Clinically suspected VAP: 15%	End of study protocol: (1) first successful weaning trial, (2) extubation, (3) death
				Microbiologically confirmed VAP, defined as clinical suspicion and positive ETS, BAL or PSB: T, 4/17; C, 5/13	Microbiologically confirmed VAP: 38%	Follow-up for an additional 72 hours after the study end point has been reached
				In-hospital mortality: T, 5/17; C, 4/13	In-hospital mortality: 31%	
van Nieuwenhoven and colleagues [20]	Adult ventilated patients with no contraindications for raised head of bed and an expected duration of ventilation >48 hours (221). Excluded: selective decontamination of the digestive tract; trauma of the pelvic region; extensive abdominal surgery; neurosurgical patients treated with 30° head elevation; patients cared for in beds without the possibility of altering backrest elevation	T (112), 45°; C (109), 10°. Backrest elevation was measured every 60 seconds by means of a transducer with pendulum. A dedicated nurse controlled patient position two or three times daily and restored backrest elevation to the randomized position when possible	Median: T, 6 (0 to 281) days; C, 6 (0 to 64) days	Clinically suspected VAP, defined as new or persistent or progressive radiographic infiltrate with at least two of the following criteria: temperature >38°C or <35°C; leucopenia or leucocytosis; positive cultures of tracheal aspirate: T, 16/112; C, 20/109	Clinically suspected VAP: 18%	End of study protocol: (1) extubation, (2) death, (3) patients were placed in a bed without the possibility to alter backrest elevation, (4) VAP
				Microbiologically confirmed pneumonia, defined as clinical suspicion and positive BAL or positive blood culture with the same microorganisms as in tracheal aspirate: T, 13/11; C, 8/109	Microbiologically confirmed VAP: 7%	Authors did not report whether there was a follow-up for an additional 72 hours after the study end point has been reached
				ICU mortality: T, 33/112; C, 33/109	ICU mortality: 30%	
				Pressure sore^b^: T, 31/112^c^; C, 33/109^c^		
				Feasibility of the allocated position^d^		

BAL, bronchoalveolar lavage; ETS, tracheobronchial aspirate; PSB, protected specimen brush; VAP, ventilator-associated pneumonia. ^a^Standard deviation (SD) could not be calculated; authors did not supply additional information. ^b^Pressure sore was staged daily to the four stages described by the National Ulcer Advisory Panel System [23]. ^c^Most patients had stage 1 or stage 2 pressure sores. ^d^The targeted 45° head of bed elevation was not reached.

**Figure 1:**
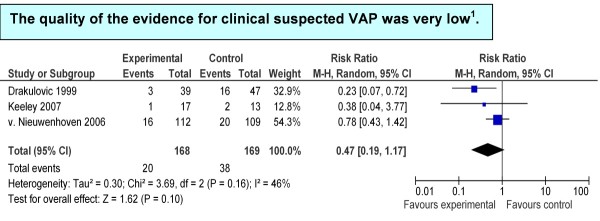
**Summary estimates of associations between treatment and control groups: clinically suspected ventilator-associated pneumonia**. **^1^**GRADE Working Group grades of evidence: high quality, further research is very unlikely to change confidence in the estimate of effect; moderate quality, further research is likely to have an important impact on confidence in the estimate of effect and may change the estimate; low quality, further research is very likely to have an important impact on confidence in the estimate of effect and is likely to change the estimate; very low quality, the estimate effect is very uncertain. CI, confidence interval; M-H, Mantel Haenszel test; VAP, ventilator-associated pneumonia.

All trials addressed clinically suspected VAP. The baseline risks for clinically suspected VAP across trials ranged from 15% [19] to 34% [8]. The pooled RR derived from all trials (337 patients) showed a benefit in favor of the 45° bed head elevation, but with wide confidence intervals (RR = 0.47, 95% CI = 0.19 to 1.17) (Figure 1).

**Figure 2:**
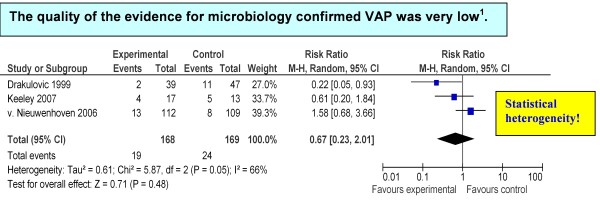
**Summary estimates of associations between treatment and control group: microbiologically confirmed ventilator-associated pneumonia**. **^1^**GRADE Working Group grades of evidence: high quality, further research is very unlikely to change confidence in the estimate of effect; moderate quality, further research is likely to have an important impact on confidence in the estimate of effect and may change the estimate; low quality, further research is very likely to have an important impact on confidence in the estimate of effect and is likely to change the estimate; very low quality, the estimate effect is very uncertain. CI, confidence interval; M-H, Mantel Haenszel test; VAP, ventilator-associated pneumonia.

Three trials reported data on microbiologically confirmed VAP. One trial included only cultures of bronchoalveolar lavages [20], whereas two trials also included cultures of tracheal aspirates [8,19]. The pooled RR (337 patients) was in favor of the 45° bed head elevation, but with wide confidence intervals (RR = 0.67, 95% CI = 0.23 to 2.01) (Figure 2).

**Figure 3:**
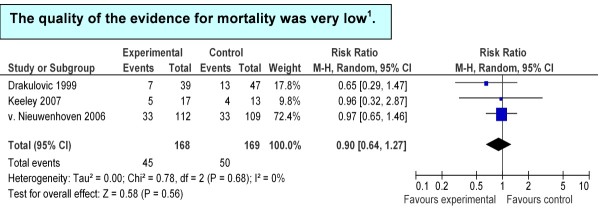
**Summary estimates of associations between treatment and control group: ICU mortality**. **^1^**GRADE Working Group grades of evidence: high quality, further research is very unlikely to change confidence in the estimate of effect; moderate quality, further research is likely to have an important impact on confidence in the estimate of effect and may change the estimate; low quality, further research is very likely to have an important impact on confidence in the estimate of effect and is likely to change the estimate; very low quality, the estimate effect is very uncertain. CI, confidence interval; M-H, Mantel Haenszel test.

Three trials reported data on mortality, including 337 patients. The pooled relative risk was in favor of the 45° bed head elevation, but with wide confidence intervals (RR = 0.90, 95% CI = 0.64 to 1.27) (Figure 3).

**Figure 4:**
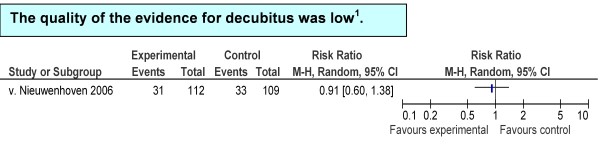
**Summary estimates of associations between treatment and control group: pressure sores**. **^1^**GRADE Working Group grades of evidence: high quality, further research is very unlikely to change confidence in the estimate of effect; moderate quality, further research is likely to have an important impact on confidence in the estimate of effect and may change the estimate; low quality, further research is very likely to have an important impact on confidence in the estimate of effect and is likely to change the estimate; very low quality, the estimate effect is very uncertain. CI, confidence interval; M-H, Mantel Haenszel test.

A single good-quality trial reported fewer decubitus ulcers in the 45° elevation group, but with wide confidence intervals (RR = 0.91, 95% CI = 0.60 to 1.38) (Figure 4).

None of the trials reported data on venous thromboembolism, hemodynamic instability, duration of mechanical ventilation, length of ICU stay, patients' comfort and patients' safety.

The estimated effects on mortality, clinically suspected VAP and microbiologically confirmed VAP were very uncertain, because the 95% CIs around the pooled effects included no effect, appreciable benefit and appreciable harm. Two RCTs exhibited serious limitations in study quality and execution: Keeley in 2007 had a nearly 50% dropout rate [19], and Drakulovic and colleagues in 1999 stopped prematurely after interim analysis showed a very large treatment effect in favor of the 45° elevation [8]. Both of these trials did not use intention-to-treat analysis and did not specify whether pneumonia assessors were blinded to treatment allocation. There was uncertainty about the directness of the evidence, because the standard care for mechanically ventilated patients with enteral feeding is not a complete horizontal position as it was in one trial [8]. Furthermore, for the microbiologically confirmed VAP outcome, meta-analysis revealed marked statistical heterogeneity that could be explained by flaws in the study design (*P *< 0.05, τ^2 ^= 0.61, *I*^2 ^= 65.9%) (Figure 2). The two poor-quality studies indicated a decreased rate of microbiologically confirmed VAP in the advantage of the 45° elevation [8,19], whereas the high-quality study indicated an increased rate of microbiologically confirmed VAP in the disadvantage of the 45° elevation [20], but all with wide 95% CIs (data not shown).

The estimated effect on decubitus was uncertain because of imprecision.

The small number of retrieved studies did not allow any subgroup meta-analysis or any assessment of publication bias.

No trials were found addressing the issue of which degree of bed head elevation does more good than harm.

### Development of recommendation by Delphi consensus process

#### Participants

Thirty-one intensive care specialists from 12 European countries were interested in participation. Twenty-seven of the 31 participants responded in the first Delphi round (87%). Twenty-four of the 27 invited experts responded in the second round (88%). Twenty-two of the 24 invited experts responded in the third round (91%). Twenty-two experts from 11 European countries thus participated in all rounds.

#### Delphi rounds

Three Delphi rounds were held. The interim results of the first and second rounds can be found in Additional file 1. Based on the scientific evidence and the results of the first and second Delphi rounds, the investigators formulated a recommendation with its rationale (Table 2). Nineteen experts (86%) agreed on the recommendation with its rationale.

**Table 2:** Recommendation on bed head elevation with its rationale

Considering that (RATIONALE):
1. based on the results of the systematic review,
• it is uncertain whether a 45° bed head elevation is effective or harmful with regard to the occurrence of clinically suspected VAP, microbiologically confirmed VAP, decubitus and mortality;
• it is unknown whether a 45° bed head elevation for 24 hours a day causes thromboembolism or hemodynamic instability;
2. maintaining a semi-upright position for 24 hours a day may cause conflict with other nursing tasks or medical interventions like insertion of intravascular catheters, providing good hygiene to the patient, prevention of decubitus, intensive physiotherapy or wound care, so that semi-upright position must be abandoned;
3. there are absolute contraindications to nursing mechanically ventilated patients in a semi-upright position - that is, patients with recent thoracic or lumbar surgery of the spine and patients with thoracic or lumbar spine injury;
4. there are a relative large number of mechanically ventilated patients with relative contraindications where caution is indicated when the patient is placed in a semi-upright position - that is, patients with hemodynamic instability; trauma of the pelvic region; and severe sacral decubitus;
5. besides the possible prevention of VAP,
a) semi-upright position of ventilated patients,
• might improve oxygenation and ventilation;
• decreases facial edema;
b) semi-upright position of awake ventilated patients,
• might promote easier communication between patients and relatives or staff, better orientation in the room and more effective coughing;
6.
• semi-uptight position of ventilated patients interferes with the prevention of decubitus - that is, changing position frequently;
• patients glide away to the foot end of the bed when using anti-decubitus mattresses;
7. the wish of awake patients to change body position regularly should be respected.
8. the intervention is no cost;
European experts in intensive medicine CONCLUDE that the recommendation should not be compelling, because the prevention of VAP is uncertain and the balance between benefits and harms is unknown, and maintaining semi-upright position interferes with other nursing tasks or with medical interventions.
The experts RECOMMEND to elevate the head of the bed of mechanically ventilated patients to a 20 to 45° position and preferably in a ≥30° position as long as it is does not pose risks and conflicts with other nursing tasks, medical interventions or with patients' wishes.

VAP: ventilator-associated pneumonia.

## Discussion

A group of 22 experts in intensive care medicine from 11 European countries recommends elevating the head of the bed for mechanically ventilated patients to a 20 to 45° position and preferably to a ≥30° position as long as it does not pose risks or conflicts with other nursing tasks, medical interventions or patients' wishes. This recommendation was based on the results of a systematic review conducted by a Dutch-German review group and the considerations brought up by the European expert panel.

Three of the 22 experts disagreed with the recommendation and its rationale. One expert was of the opinion that no recommendation should be given because of the low quality of evidence and the lack of data on potential adverse effects and feasibility. The other two experts had some additional comments on the rationale that would, however, not have substantially changed the recommendation.

The systematic review showed that it is uncertain whether a 45° bed head elevation is effective or harmful with regard to the occurrence of clinically suspected VAP, microbiologically confirmed VAP, decubitus and mortality, and that it is unknown whether a 45° bed head elevation for 24 hours a day causes thromboembolism or hemodynamic instability. In the trial by van Nieuwenhoven and colleagues, the target elevation of 45° could not be reached [20]. Grap and colleagues in 2005 also found that the mean backrest elevation was consistently lower than the recommended 30 to 45° [21]. They found an average backrest elevation of merely 21.7° in ventilated patients [6,21]. Because the desirable position for ventilated patients depends on nursing tasks, medical interventions and patients' wishes, maintaining a certain elevation for 24 hours a day is not feasible. The RCTs and the inclusion of bed head elevation in the ventilator bundle suggest to the relative outsider that a horizontal position is the standard in ventilated patients and that changing the position to a semi-upright position requires great changes in intensive care. The consultation with experienced physicians in intensive care medicine learned that some bed head elevation is common for most patients, suggesting that the trials used artificial controls. No trial will be able to replicate exact clinical practice and *vice versa*. Furthermore, modern ICUs are so complex that investigating bed head elevations in all ventilated patients or extrapolating the results to all ventilated patients is not possible. Perhaps researchers should not want to investigate bed head elevation policies at all. In any case, future research should be limited to specific ICU subgroups.

In 2009 a meta-analysis was published on the impact of patient position on the incidence of VAP [22]. This meta-analysis found that a 45° position significantly lowers the incidence of clinically diagnosed VAP compared with supine patients. Their conclusion is different from our conclusion. The main reasons are that in the previous meta-analysis the benefits and harms of a 45° position were not addressed; the overall quality of evidence by considering the items study quality and execution, inconsistency, indirectness, imprecision and publication bias were not assessed; and in their calculations of clinically diagnosed VAP, the authors pooled the data for clinically suspected VAP [8,20] with those for overall VAP [19]. We recalculated the overall incidence of VAP (clinically suspected VAP plus microbiologically confirmed VAP) using a random-effects model and did not find a significantly lower incidence of clinically diagnosed VAP.

The Ventilator Bundle of the Institute for Health Care Improvement is a series of five interventions related to ventilator care that, when implemented together, will achieve significantly better outcomes than each single intervention. Bed head elevation is one of the above components. Only patients with all five elements of the ventilator bundle in place are recorded as complying with the ventilator bundle. We would expect that bundles contain only interventions that could always be executed and that were strongly recommended. The European expert group found that the evidence was too weak, however, and that there were too many disadvantages of semi-upright positioning to formulate a strong recommendation. As such, we question whether compliance with the VAP bundle should depend on the adherence to this single item.

It is conceivable that the selected participants were not representative for all experts in intensive care medicine, but the question is whether this is desirable. In our opinion the selected participants had to be representative for the mean expert in VAP prevention. We do not see arguments that this would not be the case.

It is important to have a large expert group when going from evidence to recommendation, because the spread of answers to some questions was very diverse. Our expert group could have been a bit smaller, because we received too many of the same types of answers.

## Conclusions

Experts have made clear that the position of a ventilated patient in bed depends on many determinants. Given the scientific uncertainty about the benefits and harms of a semi-upright position, this position can therefore only be recommended as the preferred position with the necessary restrictions. The approach we have taken to develop this recommendation explicits both the scientific evidence and the arguments beyond this evidence that underpin the recommendation. The method is well applicable to develop recommendations that can count on broad support over the borders of countries and continents.

## Key messages

• The systematic review showed that it is uncertain whether a 45° bed head elevation is effective or harmful with regard to the occurrence of clinically suspected VAP, microbiologically confirmed VAP, decubitus and mortality, and that it is unknown whether a 45° bed head elevation for 24 hours a day causes thromboembolism or hemodynamic instability.

• Maintaining a certain elevation 24 hours a day is not feasible, because the desirable position of ventilated patients depends on nursing tasks, medical interventions and patients' wishes.

• The trials investigating bed head elevation used artificial controls.

• A semi-upright position can only be recommended as the preferred position with the necessary restrictions. Whether compliance with the VAP bundle should depend on adherence to this single item is therefore questioned.

## Abbreviations

CI: confidence interval; ICU: intensive care unit; RCT: randomized controlled trial; RR: relative risk; VAP: ventilator-associated pneumonia.

## Competing interests

The authors declare that they have no competing interests.

## Authors' contributions

All authors have contributed substantially to the submitted work and have read and approved the final manuscript. With regard to the review: BSN-W and AK selected the trials; BSN-W, PG, AK, RPV, JCW, and PJvdB extracted the data; and BSN-W analyzed the data and wrote the manuscript. With regard to the Delphi rounds: BSN-W and PJvdB conducted the Delphi rounds; all experts responded in all three rounds; and BSN-W wrote the manuscript. All authors read and approved the final manuscript.

## Supplementary Material

Additional file 1 **Bed head elevation study additional information**. File containing the complete search strategy, the flow diagram of reviewed articles, and more detailed information on the results of the first and second Delphi rounds. Click here for file
