# Supplementary material for: An evidence-based recommendation on bed head elevation for mechanically ventilated patients
Source: Crit Care. 2011 Apr 11;15(2):R111. doi: 10.1186/cc10135 (PMC3219392; doi:10.1186/cc10135)
Supplement: Additional file 1 — Bed head elevation study additional information. File containing the complete search strategy, the flow diagram of reviewed articles, and more detailed information on the results of the first and second Delphi rounds. [file cc10135-S1.DOC]

**An evidence - based recommendation on bed head elevation for mechanically ventilated patients**

Niël-Weise BS, Gastmeier P, Kola A, Vonberg RP, Wille JC, van den Broek PJ, on behalf of the bed head elevation study group ^see appendix^

**Search strategy**

((backrest elevation) OR (semi recumbent) OR (semi-recumbent) OR (semirecumbent)) AND ((randomized controlled trial[pt] OR controlled clinical trial[pt] OR randomized controlled trials[mh] OR random allocation[mh] OR double-blind method[mh] OR single-blind method[mh] OR clinical trial[pt] OR clinical trials[mh] OR "clinical trial"[tw] OR ((singl*[tw] OR doubl*[tw] OR trebl*[tw] OR tripl*[tw]) AND (mask*[tw] OR blind*[tw])) OR "latin square"[tw] OR placebos[mh] OR placebo*[tw] OR random*[tw] OR research design[mh:noexp] OR comparative study[mh] OR evaluation studies[mh] OR follow-up studies[mh] OR prospective studies[mh] OR cross-over studies[mh] OR control*[tw] OR prospectiv*[tw] OR volunteer*[tw]) NOT (animal[mh] NOT human[mh]))

**Flow diagram of reviewed articles**

208 titles and abstracts identified and screened. (The initial search identified 199 articles from PubMed and 68 from Cochrane Central Register. The two databases were merged and duplicates were deleted by using Reference Manager.)

not relevant (n = 205)

3 trials retrieved in full text

0 extra papers retrieved in full text after screening the references of the 3 trials

0 papers excluded

3 RCTs included in systematic review

**Results of the first Delphi round**

The participants found that the scientific evidence of a semi-upright position of 45 degrees was too weak to formulate a strong recommendation worded in terms like ‘do’ or ‘should’. The participants found that a semi-upright position 24 hours a day is not possible, because the position of ventilated patients depends upon medical and nursing factors, for example insertion of intravascular catheters, providing good hygiene to the patient, prevention of decubitus, physiotherapy or wound care.

Participants found that keeping patients in a semi-upright position is a simple intervention, and that feasibility is only becoming problematic when a 45 degrees position is aimed for 24 hours a day or if one wishes to know the angle at any time. However, the participants found that no trial would be able to replicate exact clinical practice and, therefore, the evidence and the recommendation derived from it should be interpreted pragmatically. There was no need to measure adherence to the preferred angle routinely.

Extrapolation of the scientific evidence was questioned, because the RCTs excluded many “normal” ICU patients and used rather artificial control groups which did not represent everyday intensive care.

The participants found that the fact that bed head elevation is a component of the ventilator bundle was no reason to bear in mind when formulating the recommendation; and the other way round they stated that if they did not recommend bed head elevation it would not automatically mean that bed head elevation could not be a component of a ventilator bundle.

**Results of the second Delphi round**

All considerations yielded by the first round were grouped by the investigators into six categories, i.e. other indications for semi-upright position, contra-indications for semi-upright position, additional benefits apart from reduction of VAP, drawbacks of a semi-upright position, patients’ preferences and costs. The participants were asked to indicate whether they agreed or disagreed with each of the considerations as described above and to add considerations they thought were missing. The participants were also asked which angle of bed head elevation they would prefer in a recommendation and whether they preferred the phrasing ‘routinely’ or ‘preferably’ in the recommendation.

The participants indicated the difference between absolute and relative contra-indications. Absolute contra-indications were recent thoracic or lumbar spinal surgery or injury. Caution was indicated in patients with relative contra-indications for semi-upright position, i.e. patients with hemodynamic instability, pelvic trauma and severe sacral bedsores.

According to the participants semi-upright position might improve oxygenation and ventilation and decrease facial edema. Furthermore, semi-upright position of awake patients might be in favor of easier communication with relatives and staff, better spatial orientation and more effective coughing.

As drawbacks of a semi-upright position participants mentioned the facts that patients glide away to the foot end of the bed when using anti-decubitus mattresses, and interference with prevention measures for decubitus.

Participants’ experiences were that awake patients prefer to change body position frequently.

Most of the experts chose an angle with a broad range of degrees rather than a single degree. The phrasing ‘routinely’ and ‘preferably’ were insufficient discriminative, because the experts interpreted these terms differently.

Semi-upright positioning was thought to be a cheap intervention.
